# Changes in general practice use and costs with COVID-19 and telehealth initiatives: analysis of Australian whole-population linked data

**DOI:** 10.3399/BJGP.2022.0351

**Published:** 2023-02-21

**Authors:** Danielle C Butler, Grace Joshy, Kirsty A Douglas, Muhammad Shahdaat Bin Sayeed, Jennifer Welsh, Angus Douglas, Rosemary J Korda, The ANU Telehealth in Primary Care Study team

**Affiliations:** National Centre for Epidemiology and Population Health, Research School of Population Health, Australian National University, Canberra.; National Centre for Epidemiology and Population Health, Research School of Population Health, Australian National University, Canberra.; Academic Unit of General Practice, Australian National University, Canberra.; National Centre for Epidemiology and Population Health, Research School of Population Health, Australian National University, Canberra.; National Centre for Epidemiology and Population Health, Research School of Population Health, Australian National University, Canberra.; National Centre for Epidemiology and Population Health, Research School of Population Health, Australian National University, Canberra.; National Centre for Epidemiology and Population Health, Research School of Population Health, Australian National University, Canberra.

**Keywords:** COVID-19, linked data, out-of-pocket costs, primary care, telehealth

## Abstract

**Background:**

In response to the COVID-19 pandemic, general practice in Australia underwent a rapid transition, including the roll-out of population-wide telehealth, with uncertain impacts on GP use and costs.

**Aim:**

To describe how use and costs of GP services changed in 2020 — following the COVID-19 pandemic and introduction of telehealth — compared with 2019, and how this varied across population subgroups.

**Design and setting:**

Linked-data analysis of whole-population data for Australia.

**Method:**

Multi-Agency Data Integration Project data for ∼19 million individuals from the 2016 census were linked to Medicare data for 2019–2020. Regression models were used to compare age- and sex-adjusted GP use and out-of-pocket costs over time, overall, and by sociodemographic characteristics.

**Results:**

Of the population, 85.5% visited a GP in Q2–Q4 2020, compared with 89.5% in the same period of 2019. The mean number of face-to-face GP services per quarter declined, while telehealth services increased; overall use of GP services in Q4 2020 was similar to, or higher than, that of Q4 2019 for most groups. The proportion of total GP services by telehealth stabilised at 23.5% in Q4 2020. However, individuals aged 3–14 years, ≥70 years, and those with limited English proficiency used fewer GP services in 2020 compared with 2019, with a lower proportion by telehealth, compared with the rest of the population. Mean out-of-pocket costs per service were lower across all subgroups in 2020 compared with 2019.

**Conclusion:**

The introduction of widespread telehealth maintained the use of GP services during the COVID-19 pandemic and minimised out-of-pocket costs, but not for all population subgroups.

## INTRODUCTION

During the COVID-19 pandemic, health systems around the world implemented major telehealth initiatives to ensure access to primary care while minimising disease transmission.^1^^,^^2^ In March 2020, Australia introduced new telehealth items^3^ to fund primary care teleconsultations (by telephone and video) for the whole population through Medicare, the country’s universal health-insurance scheme. Little is known of the potential impact on individual use and costs of primary care, and whether certain population subgroups were differentially affected.

Prior to the pandemic, funding for telehealth in Australia was limited to certain jurisdictions or select specialist services,^4^ with telehealth services accounting for <1% of Medicare-subsidised consultations.^5^ However, just 2 months after the introduction of the new telehealth items, telehealth consultations constituted 36% of all GP consultations (97% by audio^5^), stabilising over the remainder of 2020–2021 to ∼20% of services.^6^ Similar patterns of use have been observed internationally.^7^^–^^10^

Despite high levels of overall use of telehealth, international data have shown that uptake is not necessarily even across population groups, potentially exacerbating pre-existing inequalities in access to care, for example, in the US, children, adolescents, older people, those on low incomes, and ethnically and linguistically diverse groups have lower uptake of telehealth.^7^^,^^11^^,^^12^ Data from primary care clinics limited to specific jurisdictions, covering ∼30% of the total Australian population, have shown that older people, males, and people living in socioeconomically disadvantaged areas have lower uptake of telehealth.^13^ Few studies have examined changes in healthcare costs since the large-scale introduction of telehealth,^11^^,^^14^^,^^15^ and none, to the authors’ knowledge, have investigated whether this varies across population subgroups.

Until October 2020, Medicare-subsidised telehealth services introduced in Australia during the COVID-19 pandemic were required to be provided without cost to the patient, but how the average costs of GP services changed in relation to this is unknown.

In this study, the authors examined how individual use of GP services and associated costs changed in the latter part of 2020 — following the pandemic and introduction of population-wide telehealth — compared with 2019, particularly for groups that were medically underserved or those known to be at risk of severe disease.

## METHOD

### Data sources and study population

Data were taken from the Multi-Agency Data Integration Project (MADIP), a secure data asset combining information on health, education, government payments, personal income tax, and population demographics (including the census). Underpinning MADIP data is the Person Linkage Spine (the Spine); this is used to create a person-level identification key by linking data from three administrative databases — Medicare Consumer Directory (records of those covered by Medicare), Department of Social Services Data, and the Personal Income Tax database — resulting in virtually complete coverage of the resident population.^16^ High population coverage enables high-quality linkages of other datasets to the Spine.

**Table:** How this fits in

In response to the COVID-19 pandemic, major telehealth initiatives were implemented to ensure access to primary care while minimising disease transmission. Routinely collected whole-population data from Australia showed that the introduction of telehealth during the pandemic largely maintained use of GP services while minimising costs to patients. However, compared with pre-pandemic levels, GP use was lower among individuals aged 3–14 years, ≥70 years, and those not proficient in English — although these age groups also saw the greatest reduction in out-of-pocket costs per service. As telehealth initiatives are integrated into standard GP care, it is vital to ensure that telehealth is designed and funded to support these groups and the ongoing financial viability of practices.

Linkage was performed by the Australian Bureau of Statistics (ABS), the accredited integrating authority for this asset. For this study, the authors used the 2016 Census of Population and Housing linked to Medicare Benefits Schedule (MBS) (1 January 2019–31 December 2020) and Death Registrations (up to 2019) data. Linkage was performed using deterministic and probabilistic linking methods, using name, date of birth, address, and sex; linkage rates were 92% for census data and 97% for deaths.^16^ A direct link exists between MBS data and the Spine. The scope of the 2016 census was usual residents of Australia on the night of 9 August 2016 living in private and non-private dwellings.^17^ It had an estimated person response rate of 94.8%, with some variation in response by ethnicity and location.^18^ MBS data contain information relating to claims for medical services that are reimbursed under Medicare, including visits to GPs and other doctors outside of a hospital (identified by specific MBS item numbers). Death Registrations data contain information on month and year of death for all deaths registered in Australia; for this project, data were available for the 2016– 2019 calendar years.^19^

The study population included individuals with a 2016 census record that linked to the Spine, who had at least one MBS service claim in 2019/2020; those who had a death recorded before the end of 2019 (latest available death data) were excluded.

### Variables

Based on MBS services claimed in 2019 and 2020, the following outcome variables were derived:

- any use of GP services (yes/no) and any use of telehealth services (yes/no) in Q2–Q4 of 2020 (that is, following the introduction of telehealth items) and in Q2–Q4 of 2019;
- number of face-to-face, telehealth, and total GP services by quarter for 2019 and 2020; and
- out-of-pocket cost per face-to-face, telehealth, and total GP services by quarter for 2019 and 2020 (included MBS item numbers are given in Supplementary Table S1).

Census data were used to measure characteristics of the study population, including those that were markers of being medically at high risk of poor COVID-19-related outcomes, and other priority groups for telehealth initiatives, for example, older age and low income (Table 1).

**Table 1.** Characteristics of study population

**Characteristic**	***n***	**%**
**Total population^a^**	19 116 734	100

**Age at 1 Jan 2019, years**		
3–14	3 002 936	15.7
15–24	2 305 386	12.1
25–44	5 141 525	26.9
45–69	6 210 584	32.5
≥70	2 456 303	12.8
Missing/not applicable	0	0.0

**Sex**		
Male	9 163 494	47.9
Female	9 953 240	52.1
Missing/not applicable	0	0.0

**Education^b^**		
University degree	3 423 537	26.0
High school Year 12 or diploma/certificate	6 080 518	46.1
No high school Year 12 or diploma/certificate	3 369 575	25.6
Missing/not applicable	305 489	2.3

**Income, A$**		
≥104 000	1 785 626	9.3
65 000–<104 000	3 622 996	19.0
26 000–<65 000	7 899 299	41.3
1–<26 000	3 330 954	17.4
Missing/not applicable	2 477 859	13.0^c^

**Region**		
Metropolitan	13 687 690	71.6
Inner regional	3 481 223	18.2
Outer regional/remote	1 887 422	9.9
Missing/not applicable	60 399	0.3

**Marital status^d^**		
Partnered	9 113 613	61.6
Single	5 675 997	38.4
Missing/not applicable	0	0.0

**English proficiency^d^**		
Proficient	14 174 751	95.8
Not proficient	464 316	3.1
Missing/not applicable	150 543	1.0

**Employment status^d^**		
Employed	9 245 254	62.5
Unemployed/underemployed	5 360 630	36.2
Missing/not applicable	183 726	1.2

a *Total 2016 Census population, after study exclusions.*

b *Only those aged ≥25 years at census were included in the analysis (*n *= 13 179 119).*

c *Includes response not applicable, nil reported, and partial income reported.*

d *Only those aged ≥18 years at census were included in the analysis (* n *= 14 789 610).*

### Analysis

The proportion of people using services in Q2–Q4 of 2019 and in Q2–Q4 of 2020 were calculated, along with the mean number of services per person per quarter in 2019 and 2020, and the mean out-of-pocket costs per service per quarter in 2019 and 2020; this was done for the total sample and separately by population subgroup. Regression models were used to adjust for age (in 10-year groups) and sex, logistic regression was used to estimate the proportion of patients using services, zero-inflated negative binomial regression was used to estimate the mean number of services, and generalised linear models with a gamma distribution and logit link function were used to estimate mean out-of-pocket costs per service.

To describe changes over time, the authors calculated and plotted the adjusted mean number of services and the ratio of the adjusted mean number of services in 2020 versus 2019 by quarter; this was done for total GP services and, separately, for face-to-face and telehealth services. The same was done for out-of-pocket costs per service. The proportion of GP services by telehealth by quarter in 2020 was also plotted.

In supplementary analyses, the analyses were repeated separately for the state of Victoria (given greater community transmission of the virus in this state and more stringent public-health measures compared with the remainder of Australia) and the remainder of Australia. In sensitivity analyses, the analyses were repeated to include those who died — those participants may differ from those who did not die in their sociodemographic and health risk profile.

Stata (version 15.1) was used for all analyses, completed in DataLab, a secure remote-access computer facility for data analysis compiled and managed by the ABS.

## RESULTS

### Sample

After excluding census participants whose data did not link to the Spine (*n* = 2 650 356), who died or had invalid death dates (date of death before date of census or MBS service) (*n* = 279 654), or did not use an MBS service in 2019 or 2020 (*n* = 1 636 280), the final study population included 19 116 734 individuals (80.6% of the total census population of 23 717 418). Characteristics of the study population are shown in Table 1.

### Any use of GP services

Overall, 85.5% of people saw a GP in Q2–Q4 of 2020, compared with 89.5% in the same period in 2019. In both periods, these proportions varied across population subgroups; the variations were consistent with those typically found relating to health-service use — namely, with service use higher among females, older age groups, and disadvantaged groups (Table 2).

**Table 2.** Persons using GP Medicare Benefits Schedule services and telehealth

**Characteristic**	**Proportion using GP services, % (95% CI)**			**Proportion using at least one GP telehealth service, % (95% CI)**

	**Q2–Q4 2019**	**Q2–Q4 2020**	**Change**	**Q2–Q4 2020**
**Total**	89.5 (89.5 to 89.5)	85.5 (85.4 to 85.5)	−4.0	48.7 (48.6 to 48.7)

**Age at Jan 1 2019, years^a^**				
3–14	85.3 (85.2 to 85.3)	75.9 (75.9 to 76.0)	−9.4	30.8 (30.8 to 30.9)
15–24	85.2 (85.2 to 85.3)	80.7 (80.7 to 80.8)	−4.5	41.8 (41.7 to 41.9)
25–44	87.8 (87.8 to 87.8)	84.1 (84.1 to 84.2)	−3.7	49.0 (48.9 to 49.0)
45–69	91.5 (91.5 to 91.6)	90.0 (90.0 to 90.0)	−1.5	53.8 (53.8 to 53.8)
≥70	96.8 (96.8 to 96.8)	92.6 (92.6 to 92.6)	−4.2	62.4 (62.4 to 62.5)

**Sex^a^**				
Male	86.8 (86.8 to 86.8)	82.3 (82.2 to 82.3)	−4.5	42.6 (42.6 to 42.6)
Female	92.0 (92.0 to 92.0)	88.4 (88.4 to 88.5)	−3.6	54.3 (54.2 to 54.3)

**Education^b^**				
University degree	90.6 (90.6 to 90.7)	87.6 (87.6 to 87.7)	−3.0	54.0 (53.9 to 54.0)
High school Year 12 or diploma/certificate	91.5 (91.5 to 91.5)	89.0 (89.0 to 89.0)	−2.5	54.2 (54.2 to 54.3)
No high school Year 12 or diploma/certificate	92.0 (91.9 to 92.0)	89.4 (89.4 to 89.4)	−2.6	53.6 (53.6 to 53.7)

**Income, A$**				
≥104 000	88.6 (88.5 to 88.6)	84.6 (84.6 to 84.7)	−4.0	48.1 (48.1 to 48.2)
65 000–<104 000	89.6 (89.6 to 89.6)	85.6 (85.6 to 85.6)	−4.0	49.1 (49.1 to 49.2)
26 000–<65 000	89.7 (89.6 to 89.7)	85.7 (85.7 to 85.7)	−4.0	48.9 (48.9 to 49.0)
1–<26 000	90.0 (89.9 to 90.0)	85.9 (85.8 to 85.9)	−4.1	48.6 (48.6 to 48.7)

**Region**				
Metropolitan	90.1 (90.1 to 90.2)	85.9 (85.9 to 85.9)	−4.2	49.6 (49.5 to 49.6)
Inner regional	88.3 (88.3 to 88.3)	84.8 (84.8 to 84.9)	−3.5	49.4 (49.4 to 49.5)
Outer regional/remote	86.8 (86.8 to 86.9)	83.5 (83.4 to 83.5)	−3.3	40.9 (40.8 to 40.9)

**Marital status^c^**				
Partnered	91.1 (91.1 to 91.1)	88.5 (88.5 to 88.5)	−2.6	53.5 (53.4 to 53.5)
Single	90.5 (90.5 to 90.5)	87.2 (87.1 to 87.2)	−3.3	52.7 (52.7 to 52.8)

**English proficiency^c^**				
Proficient	90.8 (90.8 to 90.9)	88.0 (88.0 to 88.0)	−2.8	53.6 (53.6 to 53.7)
Not proficient	92.0 (91.9 to 92.1)	87.5 (87.4 to 87.6)	−4.5	40.5 (40.4 to 40.6)

**Employment status^c^**				
Employed	90.6 (90.5 to 90.6)	87.7 (87.7 to 87.8)	−2.8	52.8 (52.8 to 52.8)
Unemployed/underemployed	91.7 (91.7 to 91.8)	88.5 (88.5 to 88.6)	−3.2	54.0 (54.0 to 54.1)

a *Predicted estimated proportions are adjusted for age (in 10-year groups) and sex.*

b *Only those aged ≥25 years at census were included in the analysis (*n *= 13 179 119).*

c *Only those aged ≥18 years at census were included in the analysis (*n *= 14 789 610).*

Almost half of the population (48.7%) used GP telehealth services at least once in Q2–Q4 of 2020, up from <2% in the same period in 2019 (data not shown). Generally, groups more likely to use GP services (for example, people aged ≥70 years and females) were also more likely (than those aged <70 years and males) to use telehealth at least once. A key exception was patients not proficient in English, who were as likely to use GP services as patients proficient in English (similar proportions using GP services in 2020) but were much less likely to be users of telehealth (Table 2).

### Number of GP services

**Figure 1.**
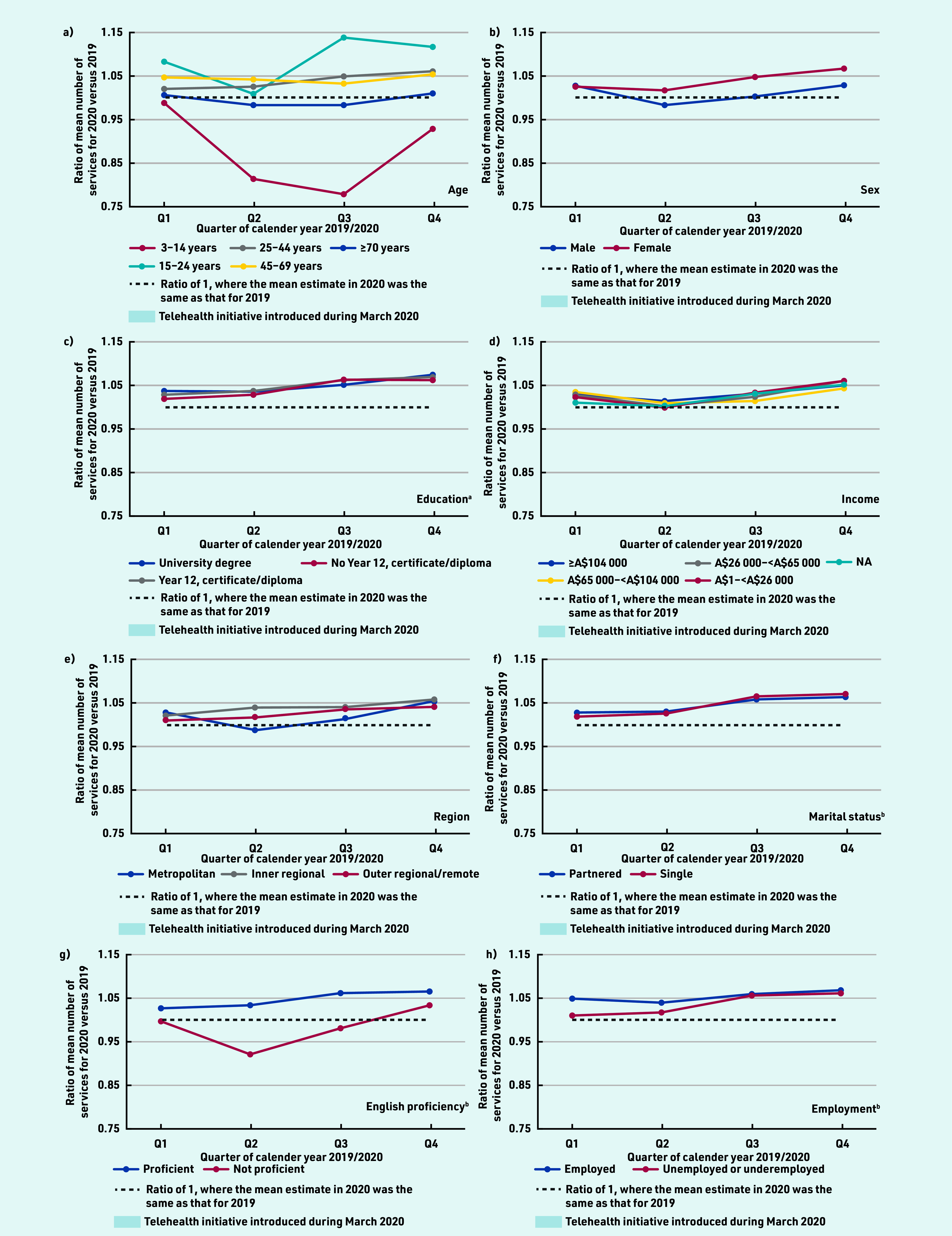
*Ratio of mean number of Medicare Benefits Schedule GP services per person for 2020 versus 2019, by quarter and sociodemographic data. Ratio of 2020 to 2019 predicted estimated mean number of services adjusted for age (10-year groups) and sex. ^a^Only those aged ≥25 years at census were included in the analysis (*n *= 13 179 119). ^b^For marital status, English proficiency, and employment status, only those aged ≥18 years at census were included in the analysis (***n** *= 14 789 610). NA = not applicable.*

The mean number of face-to-face services per person per quarter in 2020 declined after Q1 (the start of the pandemic and when wide-scale telehealth was introduced), while the mean number of telehealth services per patient increased; total GP services per patient were largely similar across quarters. This pattern was generally consistent across all sociodemographic groups examined (see Supplementary Tables S2–S4 and Supplementary Figure S1). Across most sociodemographic groups, individuals used more GP services in 2020 compared with in 2019, with ratios generally highest in Q3 and Q4; however, individuals aged 3–14 years and ≥70 years, and those with limited English proficiency used fewer GP services in 2020 than 2019 (Figure 1).

**Figure 2.**
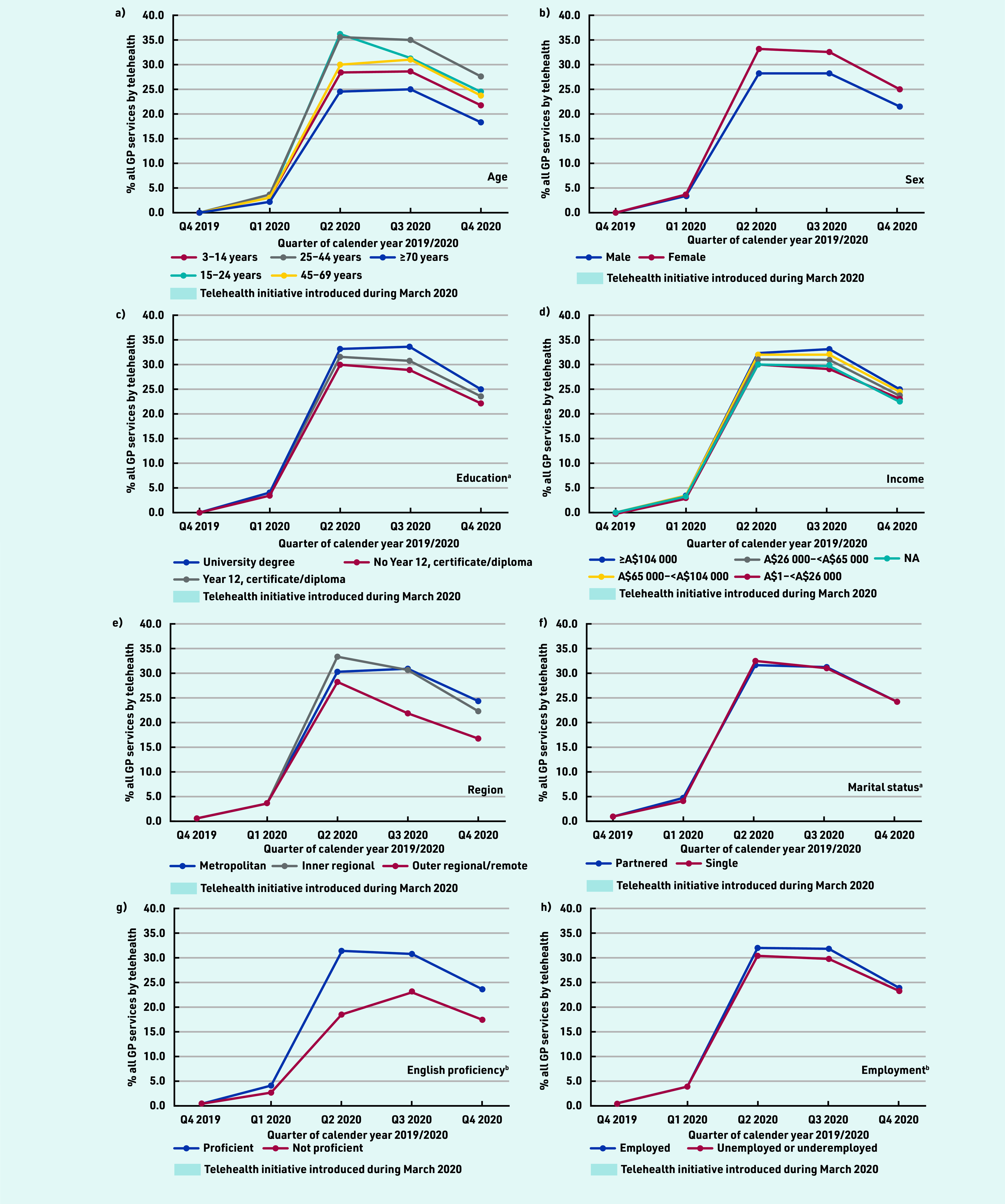
*Proportion of all GP services per person that were conducted via telehealth, by quarter and sociodemographics, 2019–2020. Proportion of predicted estimated mean number of services by telehealth, adjusted for age (10-year groups) and sex, by quarter. ^a^Only those aged ≥25 years at census were included in the analysis (*n *= 13 179 119). ^b^For marital status, English proficiency, and employment status, only those aged ≥18 years at census were included in the analysis (*n *= 14 789 610). NA = not applicable.*

Across all groups, the proportion of total services by telehealth peaked at 30%–35% before stabilising at 18%–28%, being lowest among older people, males, and those living in outer regional/remote areas or with low education, low income, and limited English proficiency (Figure 2).

### Out-of-pocket costs

**Figure 3.**
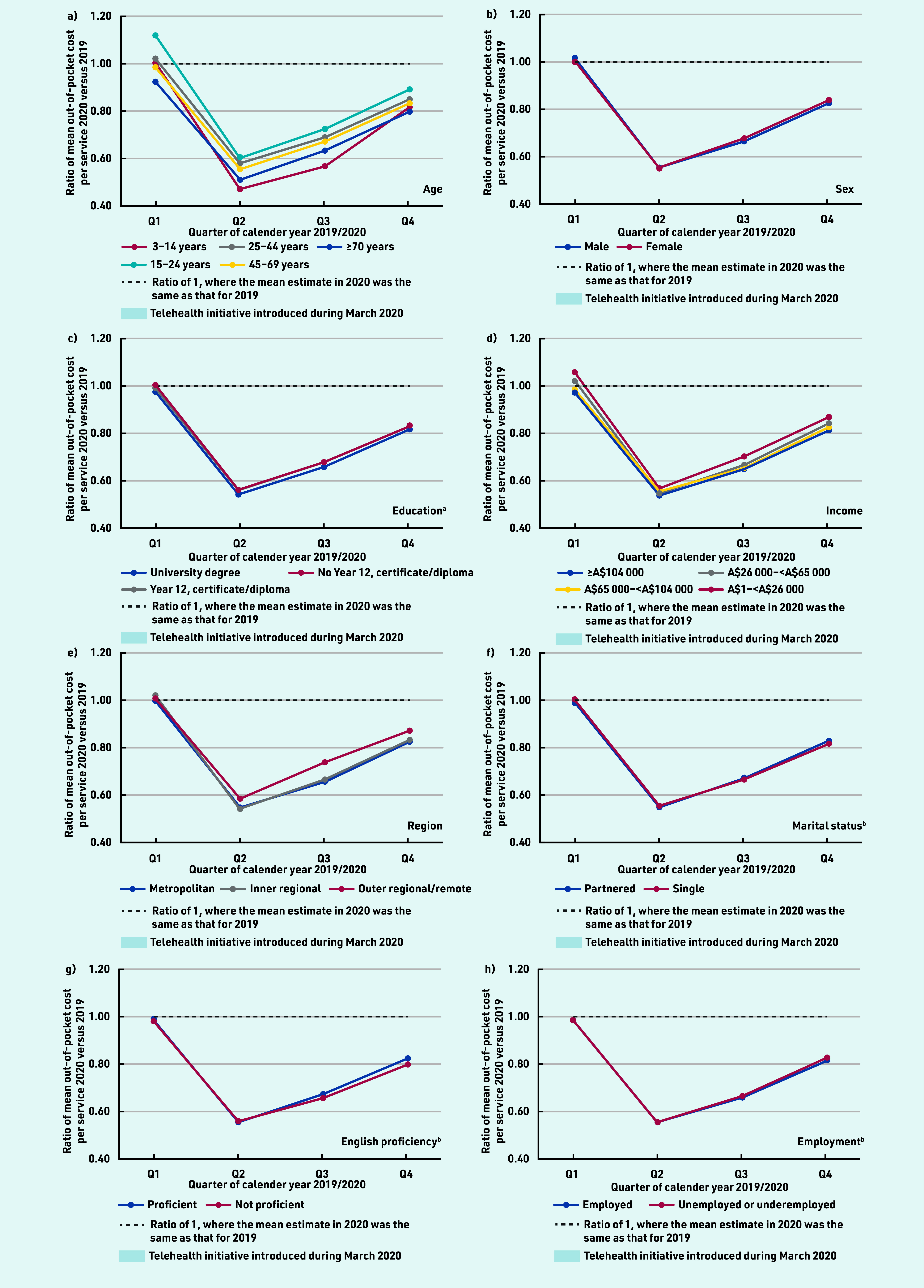
*Ratio of mean out-of-pocket cost per service for 2020 versus 2019, by quarter and sociodemographics. Ratio of 2020 to 2019 predicted estimated mean out-of-pocket cost per service adjusted for age (10-year groups) and sex. ^a^Only those aged ≥25 years at census were included in the analysis (*n *= 13 179 119). ^b^For marital status, English proficiency, and employment status, only those aged ≥18 years at census were included in the analysis (*n *= 14 789 610).*

The mean out-of-pocket cost per service in 2020 declined in Q2, from A$5 in Q1 to A$2.60 in Q2, before increasing in Q3 and Q4 but remaining below the amount observed in Q1 (see Supplementary Table S5). Across all sociodemographic groups, the mean out-of-pocket cost per service was lower in Q2–Q4 of 2020 compared with 2019, and lowest in Q2 (Figure 3, Supplementary Table S6, and Supplementary Figure S2). The largest decreases in out-of-pocket costs per service were found for individuals aged 3–14 years and ≥70 years. For telehealth services specifically, the mean out-of-pocket cost per service was very low (A$0.15 in Q1 2020) but increased over the latter part of 2020 across all groups (see Supplementary Table S7).

In supplementary and sensitivity analyses, patterns of use and costs were similar to those ascertained from the main analysis (see Supplementary Tables S8‒ S21 and Supplementary Figures S3–S6); however, when analysed separately for Victoria, the proportion of total services by telehealth peaked at 49.1% in Q2 2020, while out-of-pocket cost did not rise again until Q4 2020 (see Supplementary Figures S3b and S4).

## DISCUSSION

### Summary

In Q2–Q4 of 2020 in Australia, after the onset of the COVID-19 pandemic and following the introduction of new telehealth items, use of GP services was generally maintained while out-of-pocket costs were minimised. However, this was not the case for all groups: total GP use was markedly lower for 3–14-year-olds, older people, and people with limited English proficiency. Around one-quarter of all services were delivered by telehealth, and those who typically used more health services were also more likely to use telehealth — with the exception of those with low English proficiency. However, some key groups — often disadvantaged or medically underserved populations — had a lower proportion of services delivered via telehealth, including older people, males, people with low education or low income, those living in regional/remote areas, and those with low English proficiency. Out-of-pocket costs per service dropped substantially in the early months of the pandemic, reflecting the requirement for telehealth services to be provided with no out-of-pocket costs to the patient, with some reversal of this decline in out-of-pocket costs by the end of 2020.

### Strengths and limitations

A strength of this study is that whole-population, administrative data were used to report on the use and costs of GP services in the 9 months after the wide-scale introduction of telehealth for the Australian population. Given the direct link between the MADIP Spine and MBS data, the authors expect complete ascertainment of all outcomes among the study population.

However, there are some limitations of which it is important to be aware. Indicators of healthcare need, including health conditions, were not available in the data; for this reason, the authors relied on markers (for example, age) to indicate those medically at risk of poor health outcomes and those from traditionally underserved populations. In addition, census data were collected in August 2016; as such, a degree of change in time-varying characteristics is likely to have resulted in misclassification. Furthermore, given data availability, it was not possible to fully account for deaths to exclude those from the study population that may have been out of scope; this may have affected the results among older people, potentially explaining (at least to some degree) some of the reduction in use of GP services in 2020.

### Comparison with existing literature

As with the findings presented here, previous Australian studies have shown that overall levels of GP services were maintained in the early months of the pandemic, largely through the substitution of in-person services with telehealth;^6^^,^^20^^,^^21^ however, none of those other studies examined whether this varied across population subgroups. In other countries, with rapidly increased access to population-wide telehealth, levels of out-of-hospital care (including primary care services) were substantially reduced.^7^^–^^10^^,^^14^ Throughout 2020, Australia was still attempting to eradicate the COVID-19 virus and case numbers were comparatively low,^1^ which may explain the differences in levels of health service use with other countries. Patterns of telehealth uptake by subpopulation are similar to those reported in Australian primary care clinic data^13^ and the US.^7^^,^^10^^–^^12^

Consistent with the authors’ findings of lower uptake of telehealth among older people and those with limited English proficiency, previous research found that patients who are older, of low education, or who have poor health literacy report less satisfaction with telephone versus in-person consultations.^22^

### Implications for research and practice

The findings presented here suggest that the rapid transition in primary care to whole-population telehealth was effective in supporting access to care for the majority of the Australian population. Arguably, a need for care may have been greater in the first year of the pandemic, as COVID-19 spread and mental health conditions related to the impact of disease-control policies (such as social isolation) and financial insecurity increased; nevertheless, the findings highlight the risk that some groups — specifically, those with greater health needs, in general, but who, typically, find services harder to reach — may be missing out on care. This study identified subpopulations, including those who are underserved or medically at risk of poor COVID-19-related outcomes, that had a lower uptake of telehealth services in Q2– Q4 of 2020 (the first year of the pandemic) and the results can be used to assist GPs and local community health services to tailor how they integrate telehealth with usual clinical practice. They can also inform policy responses required to ensure that telehealth achieves the anticipated objectives.

Further work is required to understand and overcome potential barriers to use of telehealth, particularly for children and older people, and individuals with low English proficiency. The decrease in use and uptake of telehealth found in the study reported here may, for some users, be due to policy, technological, social, or other barriers, or due to pandemic-related changes in healthcare use, such as patient preference for virtual care when their provider knows them well^23^ or confidence in using virtual care technology.^24^ Similarly, providers report, as reasons for preferring in-person consultations, a lack of technology, the need for physical examination, and the inability to communicate effectively remotely.^8^^,^^25^^,^^26^ Providers report that non-verbal cues obtained during in-person consultations are more important for some groups, such as older people, those with limited socioeconomic resources, or of immigrant background,^8^ and that building rapport remotely was more challenging with patients with complex health or social situations.^27^ Variation in uptake in telehealth is also likely to depend on modality (video versus audio), which will be affected by differences in access to technology and digital literacy — at the patient, provider/practice, and area level.^28^^–^^31^ The risk of virus transmission^8^^,^^22^^,^^25^ and public-health directives on lockdowns^8^ also influence both provider and patient preferences for in-person versus virtual consultations; to what extent this varies among population subgroups is unknown. Additional exploration is needed to better understand the interplay of patient, provider/practice, and system-level factors, and whether access is equitable and according to need. Moreover, while most of the population in this study were able to access services, the impact on care quality and safety is unknown and would also warrant further investigation.

The reduction in the cost of services for all groups is likely to have offset a significant barrier to care that could have resulted from the widespread economic disruption experienced during the pandemic. Although the findings indicated that out-of-pocket costs started to increase, this had not yet reached pre-pandemic, pre-telehealth levels. Given the limited investment in general practice in the last decade,^32^^,^^33^ the ongoing integration of telehealth will need to balance affordability of services with the sustainability of general practices.

## Data

Data from the Multi-Agency Data Integration Project are available for approved projects to approved government and non-government users (https://www.abs.gov.au/websitedbs/D3310114. nsf/home/Statistical+Data+Integration+-+MADIP).
